# A practical method of drug provocation testing to prove tolerance to alternative drugs in drug hypersensitivity

**DOI:** 10.3906/sag-2005-312

**Published:** 2021-04-30

**Authors:** Ebru DAMADOĞLU, Özge ÖZTÜRK AKTAŞ, Gül KARAKAYA, Ali Fuat KALYONCU

**Affiliations:** 1 Division of Allergy and Clinical Immunology, Department of Chest Diseases, Faculty of Medicine, Hacettepe University, Ankara Turkey

**Keywords:** Adverse drug reaction, drug allergy, drug hypersensitivity, drug provocation test, provocation test

## Abstract

**Background/aim:**

Drug provocation tests (DPTs) are the gold standard method performed at the end of a stepwise approach in a drug allergy workup. Drug provocation tests are administered as a single drug on a test day. Testing more than one drug in a day is a feasible option and could be a safe and time- and manpower-saving procedure in well-selected patients. The aim of the present study was to investigate the efficacy and safety of performing a DPT with two or three alternative drugs in one test day (Hacettepe method).

**Materials and methods:**

Adult patients who were admitted with drug hypersensitivity between August 2010 and December 2016 and underwent DPTs with the described method were included. The method was based on performing DPTs with two or three different, alternative drugs on the same test day. Data was obtained from standard drug provocation test forms and from patient files.

**Results:**

A total of 1448 DPTs were performed by the Hacettepe method in 1131 patients. The reaction rate was 5.45% (n = 79), and none of the reactions were severe. The Hacettepe method saved 19.95 DPTs per month which was a considerable time savings.

**Conclusion:**

In cases of proven drug hypersensitivity reactions, performing a drug provocation test with a combination of two or three different, alternative drugs instead of one saved time and manpower and was a safe procedure. We recommend implementing this method in drug allergy workups.

## 1. Introduction

Drug provocation tests (DPTs) are the gold standard used to identify the drug responsible for hypersensitivity reactions (DHRs), and it is usually performed at the end of a stepwise approach in a drug allergy workup after in vitro and skin testing, when appropriate [1]. Providing safe drugs in cases of proven hypersensitivity is vital for patients who hesitate to take any medication, and in these cases DPT is performed to prove tolerance [1]. Nonallergist physicians and even dentists usually hesitate to prescribe medications to patients with a history of DHRs. Both European and American position papers recommend performing DPT with the primary aim of excluding DHR, emphasizing that it can also be used to confirm diagnosis or to demonstrate tolerance to an alternative drug [1–4]. 

Drug provocation tests should be performed at an allergy clinic under close observation and with one drug in a day. Drug allergy workups are time consuming procedures, and patients are scheduled for 2 to 3 test days, sometimes, even more. We hypothesized that using alternative drugs and testing 2 to 3 in a day would be a feasible option and that it would save time and manpower and remain a safe procedure in well-selected patients. 

The Hacettepe method, using double and triple tests, was previously described and has been performed in our clinic since September 2002 [5–9]. The aim of the present study was to present real-life data about the Hacettepe method and demonstrate that testing more than one, alternative drug in a single day was safe, and time and manpower saving.

## 2. Materials and methods 

This was a retrospective data review study conducted at the Hacettepe University Hospital, Department of Pulmonology, Division of Allergy and Clinical Immunology. Adult patients admitted with DHR between August 2010 and December 2016 who underwent DPT by the Hacettepe method were included in this study. Data was obtained from the standard drug provocation test forms used in our clinic and from patient files. 

The criteria required to establish the diagnosis of DHR were: a reliable clinical history with the culprit drug with at least 2 separate reactions and/or positive skin test results and/or positive DPT with the culprit drug. The drug provocation test was not performed with either the culprit drug or the Hacettepe method in cases of pregnancy or lactation; underlying cardiac, hepatic, or renal disease; severe and/or life-threatening immune-cytotoxic or anaphylactic reaction history; and patient refusal of tests with either the culprit or the method. All included patients had immediate (<1 h) or delayed (1–6 h) reactions. None of the patients with maculopapular exanthema, immune-cytotoxic reactions, or severe anaphylaxis were tested with the method. 

Written informed consent was obtained before the procedures. All provocations were performed under the close observation of at least one allergy specialist and experienced medical staff. On the test day, patients were allowed to eat a simple meal with cheese and bread. Commercially available drugs were used for testing. Drug test doses were given between 09:00 AM and 12:00 AM at 30-min intervals. When there was no reaction, the observation period ended at 17:00 PM. Reactions were treated promptly, and patients were followed until resolution of all symptoms.

**Figure 1:**
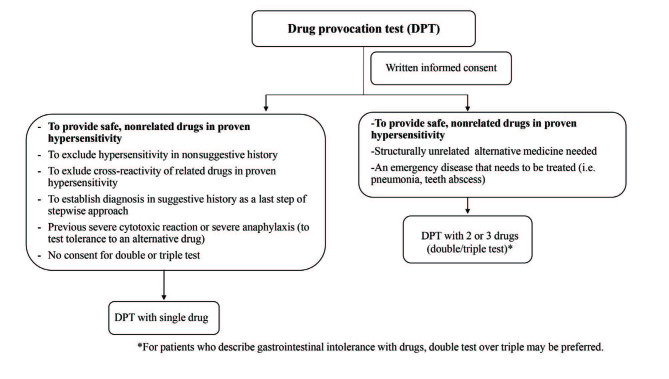
Decision to perform drug provocation test. *For patients who describe gastrointestinal intolerance with drugs, double test over triple may be preferred.

**Figure 2:**
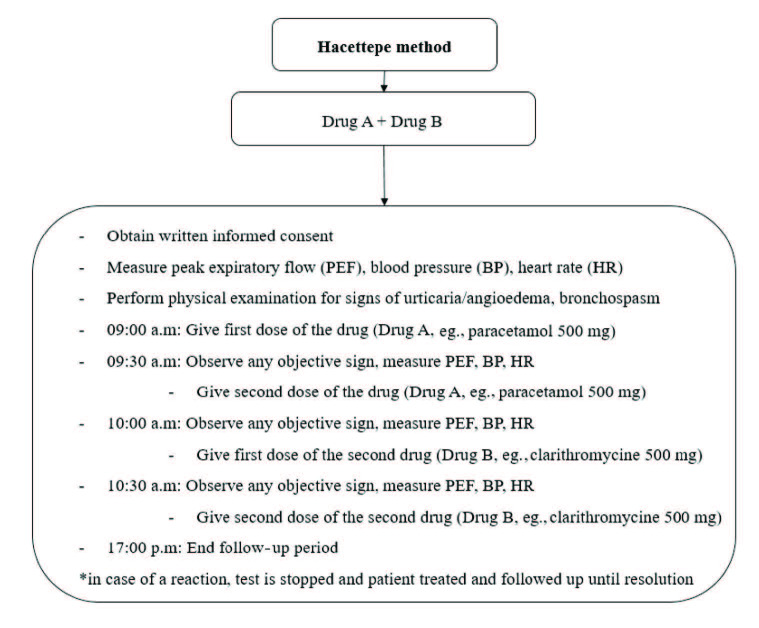
Methodology of Hacettepe method (an example for double test is given).

Figure 1 shows how decisions to perform DPT with an alternative medicine were made. Figure 2 shows the methodology of the Hacettepe method. Combinations of tested drugs were based on patient characteristics and the judgement of the allergist: e.g., two analgesics (e.g., paracetamol + meloxicam), one analgesic and 2 antibiotics (e.g., paracetamol + clarithromycin + amoxicillin – clavulanate), 2 antibiotics (e.g., ciprofloxacin + tetracycline), or other alternative combinations. Our aim was to find at least one safe antibiotic and analgesic. Each drug was given in two divided doses, making a total of 4 and 6 doses for double and triple tests, respectively. In order to provide the maximum single dose of a drug, the maximum single dose of the drug that can be given at once was divided into 2 equal doses: 500 + 500 mg for paracetamol; 7.5 + 7.5 mg for meloxicam; 100 + 100 mg for nimesulide; 500 + 500 mg for clarithromycin, ciprofloxacin, tetracycline, and amoxicillin–clavulanate; 150 + 150 mg for clindamycin; and 200 + 200 mg for moxifloxacin. 

The test result was considered positive when any objective symptom such as bronchospasm (at least a 15% drop in PEF), naso-ocular reactions, urticaria, angioedema, and/or anaphylaxis occurred. The test was considered negative when no reaction occurred at the end of the follow-up period. Reactions requiring hospitalization and/or intensive care unit admission and/or adrenalin injection, Stevens–Johnson syndrome (SJS), drug reaction with eosinophilia, and systemic symptoms (DRESS) or toxic epidermal necrolysis (TEN) were considered severe; reactions requiring a follow-up of more than one day were classified as moderate; and other reactions that resolved on test day and did not require further follow-up were classified as mild. 

In the case of an objective reaction, each drug that may have caused the reaction was tested on a separate day. However, due to the retrospective nature of the study, each and every drug in a positive double or triple test could not be tested in every patient. The patient may have refused further tests or may not have had enough time to schedule all the tests. In order to determine total time saved, the number of actual test days was extracted from the total number that would have been required, if double and/or triple tests had not been performed.

Statistical analysis was performed using SPSS for Windows version 18.0 (SPSS Inc., Chicago, IL, USA). Categorical variables were expressed as a frequency instead of versus mean ± SD, for continuous variables.

## 3. Results

Between August 2010 and December 2016, 1448 DPTs (1185 double and 263 triple tests) were performed by the Hacettepe method in 1131 patients; of these patients 800 (70.7 %) were female. Mean total test days for each patient was 2.4 ± 1.14 (range: 1–6) days. Table 1 shows the characteristics of patients who underwent at least one double or triple test. 

**Table 1:** Characteristics of 1131 patients tested with Hacettepe method.

	n (%)
Female	800 (70.7)
Age (years, mean, SD)*	40.8 ± 12.62
Total test days (mean ± SD, min-max)	2.4 ± 1.14, 1–6
Total number of double and triple test	1448 (100)
Total number of reactions in double and triple test	79 (5.45)
Double test, n (%)• Antibiotics• Analgesics• Combination (antibiotics and analgesics)	1185 (81.8)• 553 (46.7)• 600 (50.6)• 32 (2.7)
Triple test, n (%) • Antibiotics • Analgesics • Combination (antibiotics and analgesics)	263 (18.2)• 179 (68)• 78 (29.7)• 6 (2.3)

*Data available in 1090 patients.

The number of positive test results which required further testing was 79 (5.45%). The mean age of the patients who tested positive was 40.22 ± 13.27 years, and 59 (75%) of these patients were female. Table 2 shows the reaction characteristics of those 79 patients. The majority of the reactions were mild or moderate in severity, and no anaphylaxis occurred; most of the reactions were urticaria and/or angioedema (65.8%). The clinical data from 62 patients (out of 79) for accompanying atopic disease and culprit drugs were available; among these patients 43 (69.4%) had at least one accompanying atopic disease. Culprit drugs were NSAIDs, antibiotics, and both in 52 (83.9%), 1 (1.6%), and 9 (14.5%) patients, respectively. Seventy-one reactions in double or triple tests occurred when combinations of paracetamol, meloxicam, and/or nimesulide were tested. In the remaining 8, reactions occurred with a combination of antibiotics, and in 6 out of 8 of these the reaction was gastrointestinal intolerance, not hypersensitivity. 

**Table 2:** Characteristics of 79 patients that had a reaction on drug provocation test with Hacettepe method.

	n (%)
Female	59 (75)
Age (years, mean, SD)	40.22 ± 13.27
Atopic disease*• Asthma• Allergic rhinitis and/or rhinosinusitis• Chronic urticaria• None	43 (69.4)25 (40.3)35 (56.5)10 (16.1)19 (30.6)
Culprit drug*• NSAIDs• Antibiotics• NSAIDs and antibiotics	52 (83.9) 1 (1.6) 9 (14.5)
Reaction on double and triple test• NSAIDs• Antibiotics• Combination of NSAIDs and antibiotics	71 (89.9)8† (10)0
Reaction severity• Mild• Moderate• Severe	69 (88)10 (12)0
Reaction type• Gastrointestinal • Respiratory • Urticaria with/without angioedema• Isolated angioedema	7 (8.9)20 (25.3)40 (50.6)12 (15.2)

*Data available in 62 patients, †6 gastrointestinal intolerance, 2 urticaria.

In 71 patients who tested positive with NSAIDs in double and/or triple tests, further tests with these drugs were performed. A drug provocation test was performed with each single drug on a separate day in 18 (25.4%) patients, and drugs were tolerated; in 26 patients (36.6%) at least one but not all of the drugs were tested, and the tested drugs were tolerated; and in 16 patients (22.5%) the tested drugs caused a specific reaction when each drug was tested on a separate test day. A total of 11 (15.5%) patients did not accept further tests.

If double and/or triple tests had not been used and each drug had been tested on a separate day, 1516 additional test days would have been required to prove tolerance to alternative drugs. Considering the study inclusion period of 76 months (2280 days), the Hacettepe method saved 19.95 test days per month (additional test days = 1516/inclusion period = 76 months), which is a considerable amount of time.

## 4. Discussion

Although European, American, and most national guidelines define indications, contraindications, and methods of drug allergy workup, there are differences between countries in practice. Performing a DPT with the culprit drug should be different than performing it with an alternative drug. If the original reaction is severe, the indications for DPT should be carefully reviewed, the possibility of cross-reactions should be considered, and each test should be performed with a single drug. NSAID reactions may be of the cross-reactive type, and in these cases double or triple tests with 2–3 different NSAIDs, even if the tested NSAIDs are weak COX-1 inhibitors, may expose patients to increased doses and cause reactions. However, if the culprit NSAID reaction is an anaphylaxis and the patient is a single reactor (i.e. has an IgE-mediated reaction to a single NSAID or NSAID group), double and/or triple testing with alternative NSAIDs would be a safe procedure [10,11]. 

Performing a DPT with alternative drugs to prove tolerance is generally safe. Drug provocation tests are time consuming procedures that require a follow-up period after the last dose of the drug is given. There is general consensus regarding the contraindications of DPT, whereas the challenge procedure varies a great deal from one center to another [12]. If the indication for a DPT is to exclude or confirm a diagnosis of drug hypersensitivity, DPT should be performed with a single drug and, depending on the severity of the culprit reaction, the starting dose should be 1:10.000 to 1:10 of the therapeutic dose [1]. However, if the indication is to find an alternative drug, based on our experience, we recommend beginning with higher doses; instead of giving 4–5 divided doses, we recommend 2 doses.

In this real-life study featuring a considerable number of DPTs by the Hacettepe method, we showed that testing two or three different types of drugs in a single day was safe and saved time and man-power. The reaction rate was 5.6%, and most reactions were mild (88%) with no severe reactions. The Hacettepe method saved 20 test days per month, and there were fewer total test days for each patient. 

We initially described the method in 84 patients who were admitted with NSAID hypersensitivity between September 2002 and July 2004 [5]. Triple combinations of meloxicam, rofecoxib, celecoxib, benzydamine, azapropazone, codeine, and paracetamol were tested in a prospective study design. Although all positive triple tests were performed on separate days with the same drug in the same order and with the same dose, the method saved 116 test days. There were 18 reactions (21.4%), and 4 (22%) revealed no reaction when the tests were repeated using a single drug on separate days [5]. In this study, NSAID hypersensitivity was proven in all patients, the drugs tested were weak COX-1 and COX-2 inhibitors and codeine, and no severe reactions occurred. In real life, 678 double and/or triple DPTs with NSAIDs have been performed with a reaction rate of 10.5% (n: 71), which was lower than previously reported (21.4%). The possible reason for the lower reaction rate in real life was the heterogeneity of the population; some patients had hypersensitivity to drugs other than NSAIDs. 

In the second study, double and triple tests with antibiotics were performed in 15, 17, and 21 patients with antibiotic, antibiotic and NSAID, and NSAID hypersensitivity, respectively [6]. Patients were prospectively enrolled between September 2005 and December 2006. Roxithromycin, tetracycline, ciprofloxacin, ampicillin, clindamycin, and clarithromycin were used in double or triple combinations as described [6]. A total of 53 provocations were performed with 4 reactions, and 2 of the reactions did not recur when the drugs were tested one by one on separate days. However, mild and acceptable gastrointestinal side effects like nausea and vomiting were reported to be high (21.9%), more specifically with the triple test [6]. We observed that triple combinations of antibiotics frequently caused mild nausea, which does not usually require intervention with antiemetics and resolves by the end of the test day. 

The third study was designed to test combinations of antibiotics and analgesics as a triple test in multidrug hypersensitive patients, and results were satisfactory with no severe adverse effects or reactions [7]. In the present study 38 (5%) double and/or triple tests were performed as a combination of antibiotics and analgesics, and there were no reactions recorded. 

It was reported that 21% of penicillin-allergic patients may demonstrate allergy to other groups of antibiotics, namely quinolones, and previous history of an immediate hypersensitivity reaction to beta-lactam antibiotics was a strong risk factor for quinolone hypersensitivity [13]. From this point of view, it would not be a safe to suggest that the quinolone group of drugs are reliable without proving tolerance. 

Due to its retrospective design, the study has some limitations. Nevertheless, it provides important data about the application of the Hacettepe method in real life. The main limitations include potential under-recording of some of the minor reactions like nausea or nonspecific itching, and this method of DPT cannot be recommended for children before proof of safety because pharmacodynamics and drug interactions in children are different than those in adults. Another limitation was the heterogeneity of the study population. We believe that our study may provide clinicians with a different perspective for DPT. 

In conclusion, drug hypersensitivity reactions are common and of significant concern for physicians and patients. When finding a safe alternative drug for a drug hypersensitive patient is indicated, we recommend a novel DPT method. After 16 years of experience in applying the Hacettepe method of DPT, we conclude that it is a safe and time- and manpower-saving procedure and we recommend its implementation in drug allergy workups. 

## Informed consent

The study protocol was approved by the institutional review board (no. GO 17/11-12).
